# A review of multi-disciplinary decomposition research and key drivers of variation in decay

**DOI:** 10.1007/s00414-024-03222-2

**Published:** 2024-04-16

**Authors:** Donna B. McIntyre, Blake M. Dawson, Benjamin M. Long, Philip S. Barton

**Affiliations:** 1 Future Regions Research Centre, Federation University, Mount Helen, VIC 3350 Australia; 2 Graduate Research School, Federation University, Mount Helen, VIC 3350 Australia; 3 School of Environmental and Rural Science, University of New England, Armidale, NSW 2350 Australia; 4 School of Life and Environmental Sciences, Deakin University, Geelong, VIC 3216 Australia

**Keywords:** Volatile Organic compounds, Microbes, Entomology, Necrobiome

## Abstract

The decomposition of animal remains is a multifaceted process, involving ecological, biological, and chemical interactions. While the complexity is acknowledged through concepts like the necrobiome, it’s unclear if this complexity is reflected in research. Appreciation of the complexity of decomposition is crucial for identifying sources of variation in estimations of time since death in medico-legal science, as well as building broader ecological knowledge of the decomposition process. To gain insights into the extent of multidisciplinary research in the field of decomposition science, we conducted an examination of peer-reviewed literature on four key drivers of variation: volatile organic compounds, microbes, drugs/toxins, and insects. Among 650 articles, we identified their scientific discipline, driver/s of variation investigated, and year of publication. We found that 19% explored relationships between two drivers, while only 4% investigated interactions between three. None considered all four drivers. Over the past three decades, there has been a steady increase in decomposition research publications, signifying its growing importance. Most research (79%) was linked to forensic science, highlighting opportunities for interdisciplinary collaboration in decomposition science. Overall, our review underscores the need to incorporate multidisciplinary approaches and theory into contemporary decomposition research.

## Introduction

The decomposition of vertebrate remains consists of biological, chemical, and physical changes, resulting in the breakdown of large, complex organic molecules, and recycling of nutrients and energy back into ecosystems [1–6]. External and internal factors such as temperature, humidity, exposure, burial, health, diet, medical history, age, and genetics can influence the rate of decomposition and create variability in the process [7–13]. This variability is important to understand because it affects how we measure, explain, and predict decomposition rates under a broad range of scenarios, and therefore informs our capacity to explain variation in estimations of time since death, otherwise known as the post-mortem interval (PMI), within different biomes [9, 14, 15]. The community of decomposer organisms and their interactions, often referred to as the “necrobiome,” are an important source of variety that have attracted much interest. In order to integrate decomposition theory with the determination of the PMI, the necrobiome serves as a fundamental theoretical basis. It includes the biological and ecological processes and functions that are governed by bacteria, insects, and vertebrates within a broad framework that also considers their interactions with abiotic factors, soil, and the surrounding environment.

The necrobiome framework provides a foundation for understanding the relationship between multiple drivers of variation (hereafter referred to as ‘DoV’) in decomposition, which are individual components that create variability within decomposition and affect the rate of decay [16]. There are very few controlled experimental manipulations of differing components of the necrobiome model, but this is a keyway to validate the model and determine the relative roles and importance of multiple DoV. For example, a study by [17] investigated decomposition rates of wild rabbits which were either buried after exposure to insect activity, buried without exposure, kept above ground with insect excluded, or exposed to insects above ground. Their results demonstrated that insect presence was the primary agent affecting decomposition rate [17]. Another study by [18] compared insect activity and decomposition between humans and pigs, and discovered variability between insect species richness, colonisation, and decomposition rate. They theorised that these results were due to the differences in mass, diet, medical history, and microbiomes [18]. This highlights the importance of taking into consideration the variability of DoV between research models.

In another study by [19], researchers investigated the relationship between three key components (epinecrotic bacteria, volatile organic compounds (VOCs), and flies) during the first 4 days of decomposition using 75 piglet cadavers in three different forest regions. VOCs are organic molecules that are released into the surrounding environment during decomposition [20]. These compounds make up a variety of chemical classes; carboxylic acids, alcohols, aromatics, aldehydes, ketones, hydrocarbons, esters, ethers, nitrogen compounds and sulphur compounds, and originate from the community of micro-organisms within and around the carrion [21]. They play a vital role in the attraction and repulsion of insect and vertebrate scavengers to decomposing remains, serving as chemical cues that guide their search for food and colonisation sites [22, 23]. Their findings revealed dynamic changes in bacterial populations and VOC emissions during decomposition, which were influenced by factors like temperature and time but not by the specific forest region. However, the presence of flies varied both spatially and temporally.

The study highlighted a strong interdependence among these three components, primarily regulated by the temperature and time since death, as well as the specific study regions. Interestingly, this interdependence remained consistent across a gradient of forest management intensity. By examining the interactions between these components, the research contributed to a better understanding of the holistic mechanisms governing carrion community dynamics and cross-kingdom interactions, which are essential for describing food web dynamics and overall ecosystem functions [19].

In this review, we examined the peer-reviewed literature for studies of interdependent relationships between DoV in decomposition to investigate the extent of multidisciplinary perspectives in decomposition science. The complexity underpinning decomposition has been known for some time [13], and has since been built upon by the necrobiome framework [16] and recognised in the forensic literature [5]. Despite this growth in knowledge, it remains unclear how multidisciplinary perspectives have been adopted in the literature and if complexity is being incorporated into more recent studies. Our aim was to quantify how many studies examined more than one DoV, and to identify which subdisciplines tended to incorporate additional variables. We discuss our findings in light of improving understanding of decomposition and its implications for identifying factors that influence the rate of decay and forensic applications.

## Methodology

We focused on four key variables in decomposition sciences, which we considered to be significant factors associated with variation in decay: VOCs, microbes, drugs and toxins, and insects. We further divided the topic of insects into 2 separate subcategories: (1) research on the pre-appearance interval (PAI, the interval preceding insect appearance) and colonisation (oviposition), and (2) insect succession.

We then conducted a literature search to identify peer-reviewed articles related to decomposition and necrobiome. We searched databases including PubMed, Scopus, and Web of Science, using the keywords “decomposition,“, “decay”, “necrobiome,” “animal remains,“, “cadaver”, “microbes,” “insects,” and “volatile organic compounds.” These broad search terms would ensure that we started with a larger group of literature, which we could further refine.

We initially found 16,121 articles between five different topic searches targeting VOCs, microbes, insects, drugs and toxins, and PAI and oviposition. We included publications from the first instance, to end of 2022, and then screened the titles and abstracts of identified articles to exclude irrelevant studies which neglected investigation of decomposition or one of our chosen DoV. We then selected those that met further inclusion criteria: studies that investigated the relationship between DoV and their subsequent effect on decomposition and role within the necrobiome.

From the final list of selected articles (*n* = 650), we extracted the following data:

- year of publication;
- mentions or interactions of other drivers of variability (such as research, reviews or case studies which investigated or considered VOCs, microbes, insects, and toxicology) within each topic, and;
- the disciplinary focus of the research– these were decided based on whether the paper itself suggested the application of the research to a particular discipline, or if this information was missing, which journal the paper was published in. The articles were then clearly divided into categories of forensic, ecological or combination; if the article mentioned more than 1 application to a discipline.

This data allowed us to identify what research has been conducted on the interaction between DoVs, how research has shifted over time, and if the scope of the research has connected multiple disciplines.

We analysed the extracted data to identify common themes and patterns across studies, and to answer our research questions. We used a narrative synthesis approach to analyse the data, which involved summarising the findings of each study and identifying patterns and relationships between them. We presented our findings in a descriptive and organised manner, including tables and figures to illustrate key findings. We utilised R version 2023.03.1 + 446 [24] and (ggplot package [25]), with the aid of Artificial Intelligence program, Chat GPT [26], to assist in coding, and Microsoft Excel [27] to create data visualisations.

## Results

### Microbes

**Fig. 1:**
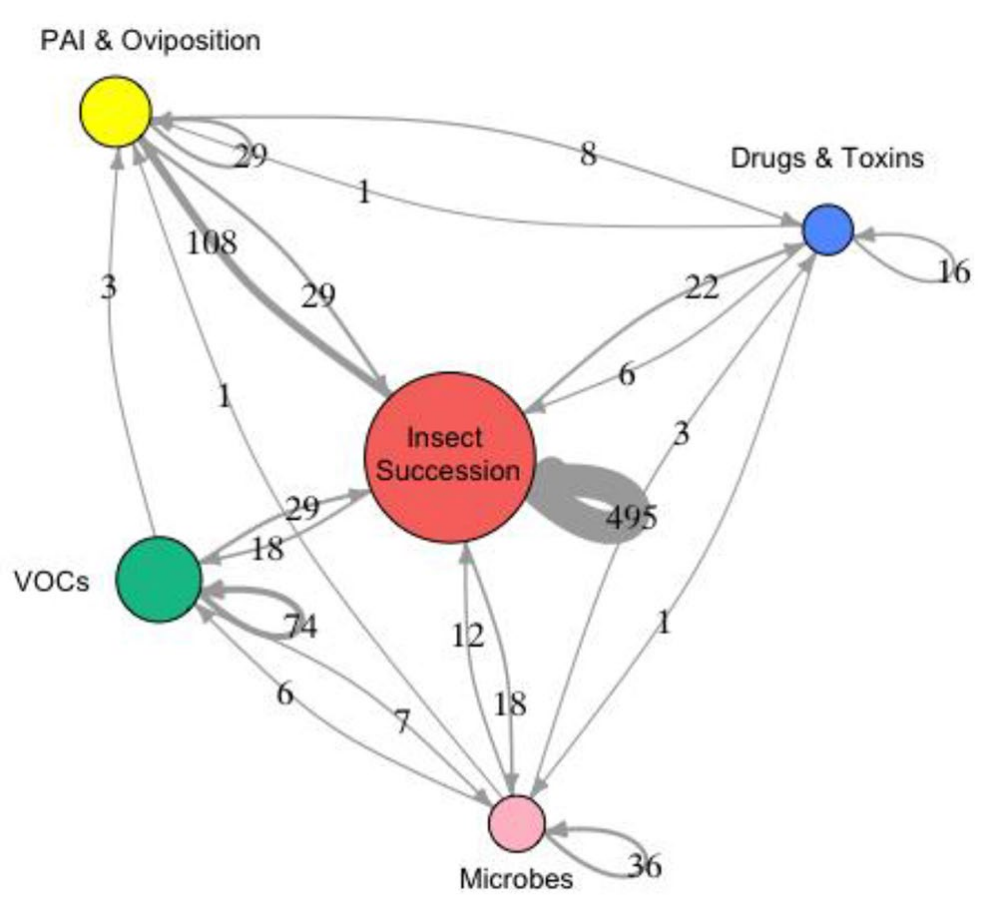
Weighted network diagram showing the number of articles reporting pairwise combinations of our topics. Line thickness is directly proportional to the number of publications which consider four or more drivers of variation between the topics. Arrows circling back depict total number of articles within each topic

We found 36 microbial-focused research articles from between 2009 and 2022; 22 articles focused on microbes only (i.e. one DoV), 10 considered the relationship between two DoV, and 10 between three. None considered or investigated the relationship between four or more DoV (Fig. 1). 12 articles discussed the role of insects in decomposition and their impact on the microbial community (i.e. two DoV), and one article emphasised the importance of considering the PAI and oviposition (i.e. two DoV). Six articles explored the relationship between VOCs, decomposition, and the microbiome (i.e. three DoV), while three articles investigated the influence of drugs (ethanol, GHB, nitrobenzodiazepines) and the toxin lead on the decomposition process involving microbes (i.e. two DoV) [28–30].

**Fig. 2:**
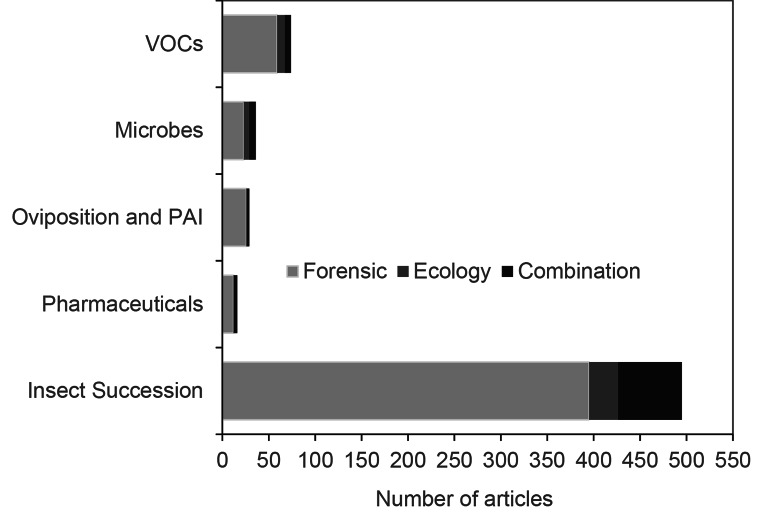
Number of research articles on five categories of drivers of variation grouped by their corresponding forensic or ecological scientific discipline

**Fig. 3:**
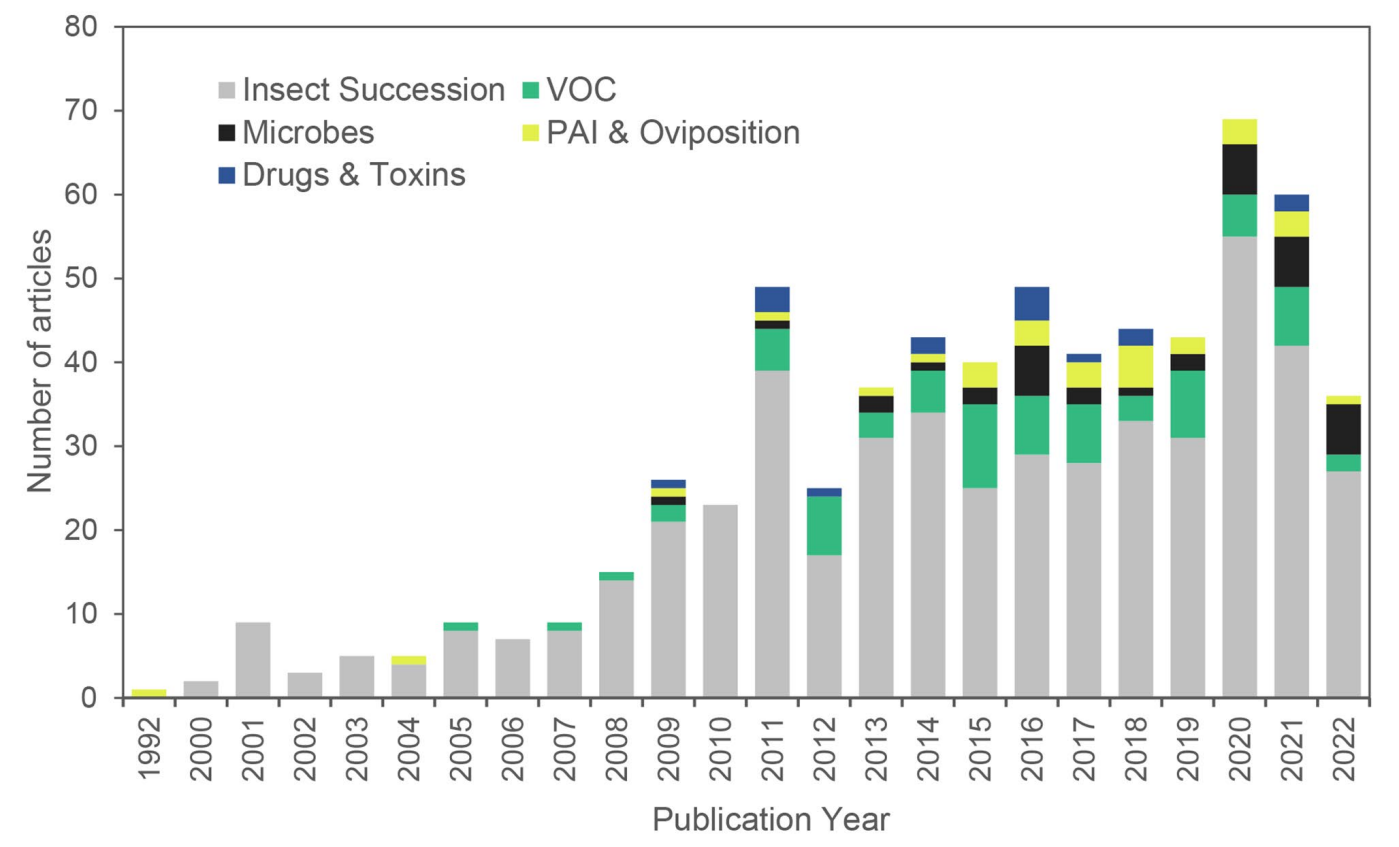
Number of research articles on five cataegories of drivers of variation over the last 30 years

We found that the majority of the microbial research articles focused on forensic science, with 23 out of 36 articles categorised in this discipline, while six articles had an ecological focus (Fig. 2). The remaining consisted of a combination of scientific disciplines. We also observed that there has been a notable increase in microbial research since 2020, with six articles published each year (Fig. 3).

### Insects (succession)

The largest review was conducted on insect succession/behaviour articles, as we found 495 research articles from between 1992 and 2022, of which 353 articles focused on entomology only (i.e. one DoV). 108 articles considered the PAI and oviposition (Fig. 1), and there was an equal amount of research articles (18) which explored the role of VOCs as well as microbes in decomposition. (i.e. three DoV). A total of 22 articles investigated the effects of various chemical toxins on decomposition, covering a wide range of drugs: drugs of dependence [31–33] malathion [34–38], pesticides and insecticides [39–41], insect repellent [42], gasoline [43–45], carbon monoxide [46, 47], alcohol [48, 49], and bleach and hydrated lime [44, 50, 51].

The majority of entomology articles focused on forensic science (395 articles), with a smaller proportion categorised as ecology or a combination of scientific disciplines (Fig. 2). There was no clear trend observed regarding the year of publication, although there was an increasing prevalence in the last decade (Fig. 3).

### Insects (PAI and oviposition)

We found 29 research articles investigating PAI and oviposition, with 21 articles focused only on PAI/Oviposition. We found eight also considered the effect of a single other DoV; drugs and toxins, but none investigated relationships with microbes or VOCs, or multiple DoV (Fig. 1). The drugs and toxins investigated in these articles included malathion [35], paraquat [52], diazepam [53], antifreeze [54], citronella and chlorpyrifos [55], hydrated lime and bleach [50].

We noted that most of these publications were within the forensic science discipline, with only two falling under a combination discipline (Fig. 2). We also observed a slow but increasing number of PAI and oviposition articles within the scientific discipline in recent years (Fig. 3).

### Volatile Organic compounds (VOCs)

We found 74 articles on VOCs, of which 44 focused only on VOCs. We found that none of the articles considered drugs or other toxins. Out of all VOC articles, 29 focused on insect involvement and effects, while three considered microbes. Only three investigated effects on PAI and oviposition (Fig. 1).

Many of the reviewed articles had a forensic science discipline (59), with eight solely within the ecology discipline – the remaining several were a combination discipline (Fig. 2). Research on decomposition VOCs was first published in 2005 peaked in 2015 with 10 articles and has gradually climbed in number of publications (Fig. 3).

### Drugs and other toxins

We found 16 articles on drugs and other toxins, of which 9 focused only on pharmaceutical substances. We found commonly abused substances were investigated, including benzodiazepines and synthetic cannabinoids [56], alcohol [49], cocaine [57], dextromethorphan and dextrorphan [58], tramadol [31], ketamine [59], and gasoline [45]; as well as key drugs and toxins such as cytotoxic chemicals and antibiotic treatments [60], clozapine [61], amitriptyline [62], strychnine and delorazepam [63]. We found a review of 39 case studies which investigated the utility of bone in detecting basic substances such as benzodiazepines, opiates, cocaine and metabolites [64].

Only six articles explored the relationship between drugs and toxins in decomposition, and insects, and only one article investigated how drugs and toxins may affect PAI and oviposition (Fig. 1).

Most (12) of these articles had a strong forensic science discipline, with a few falling into the combination category (Fig. 2). There does not appear to be any trend in publication year, with the first article being published in 2009, and the latest in 2021; the most publications occurred in 2016 with four articles (Fig. 3).

Overall, from 650 reviewed articles, only 174 (19%) investigated the effect between two drivers of variation, and 29 (4%) investigated the effect between three. None investigated the effect between four of more drivers of variation in decomposition.

## Discussion

In this review we set out to identify the extent to which the literature was multidisciplinary in existing work in decomposition science. We did this by identifying key topics of interest and providing a descriptive account detailing the consideration of multiple drivers of variation in decay, the developments within publications across the years, and multidisciplinary perspectives.

Overall, we found there was a larger focus on the role of microbes and insects in decomposition than on PAI and oviposition. The study of VOCs and the influence of drugs and toxins on decomposition was also less common. Additionally, most of the reviewed literature had a strong forensic science discipline when compared to the other disciplines (ecological/veterinary/medicine), despite the interdisciplinary nature of these fields. For example, taphonomy, entomology, botany (ecology), animal forensics and zoonotic diseases (veterinary), and forensic pathology and toxicology (medicine), are some areas where these disciplines intersect.

Despite the individual contributions of each driver of variation in decay, growing empirical evidence suggests that these factors are interconnected and influence one another. However, no research articles were found in our review that investigated the relationships between all of our key variables simultaneously. This highlights an important knowledge gap and emphasises the need for collaborative efforts within the decomposition science community [5].

### Microbes

Microbes play a significant role in decomposition, and their interactions with insects and the surrounding environment are vital factors to consider [65–67]. The limited number of articles addressing the influence of insects on the microbial community highlights the need for further exploration [14, 68]. Understanding the intricate relationship between insects and microbes during decomposition can provide insights into how changes in insect behaviour and colonisation patterns influence microbial dynamics, and vice versa. For example [69], discovered that the progression of carrion-frequenting insects is influenced by shifts in microbial communities and the release of volatiles throughout decomposition. Additionally, they observed that certain insect species require specific combinations of volatiles to accurately identify the desired stage of decomposition. Another study by [70] demonstrated that flies found on human cadavers exhibit a microbiome composition that is comparable to flies from previous studies unrelated to human cadavers. However, variations in the microbiome were observed across different seasons and different parts of the flies’ body. Their research provides evidence supporting the role of flies as a potential source of microbial transfer to the human decomposer microbiome. Another study by [71] developed a novel model of bacterial community succession, transmigration and differential gene transcription which supports the theory of predictable microbial successions after death, in response to environmental variability. These findings contribute to our understanding of the ecological processes involved in the assembly of microbial communities associated with human cadavers. Another area for research is assessing the reliability of microbial data in trace evidence research. This area holds potential for enhancing investigative methodologies and can build on initiatives such as the Human Microbiome Project [72]. It is becoming increasingly clear that insects, VOCs, and microbes play an important role in decomposition variation, and a multidisciplinary perspective is key to improving understanding in this area of decomposition science [73].

Our review revealed that most of the reviewed articles (23 out of 36) were oriented towards forensic science, highlighting the strong influence of microbial research in this field. This suggests that researchers understand the significance of the microbiome in decomposition, and how understanding these microbial communities is essential for accurate PMI estimations and determining the cause and circumstances of death [12, 28, 67, 74–76].

While forensic science dominates the microbial research landscape in decomposition science, our review also identified a smaller proportion of articles (six) with an ecological focus. This suggests that researchers recognise the broader ecological implications of microbial communities in the context of decomposition. Ecological studies explore the role of microbial communities in natural environments, including the decomposition of organic matter in various ecosystems [16, 77, 78]. By investigating the dynamics and functions of microbial populations during decomposition, these studies contribute to our understanding of ecological processes, competitive interactions, and the recycling of nutrients in ecosystems.

Our results for publications indicate a notable increase in the popularity of microbial research related to organic decomposition since 2020, with six articles published each year. This suggests a growing interest and recognition of the significant role that microbial communities play in the decomposition process. The rise in research publications in this area reflects the increasing recognition of the intricate relationship between microbes and decomposition and highlights the importance of studying microbial dynamics in understanding decomposition processes. Additionally, we are now witnessing the advent of innovative techniques which enable cheaper and more streamlined methodologies for microbiome sample processing and analysis of nucleotide sequence data. These factors combined, have likely facilitated the increase in research and publications in the field of decomposition research [79].

### Insects (succession)

Insects have long been recognised to play a major role in the decomposition process [80, 81], and will accelerate decay [17, 82, 83]. Despite the clear significance of insects in decomposition [50, 69, 84–91], our review highlights a scarcity of research which investigates the impact of key factors which can influence insect activity in decomposition, such as the influence of drugs and toxins [50, 51, 57]. Our review also noted that despite the application of insect research to various fields of science, the reviewed articles were predominantly skewed towards the forensic discipline. This finding highlights the strong focus on insects in forensic investigations of decomposition, particularly in estimating time since death and determining the circumstances surrounding human remains. While forensic entomology has gained significant attention, it is important to acknowledge the broader ecological and medical implications of insect decomposition research. In ecological studies, insects’ role in nutrient cycling, decomposition rates, and community dynamics can provide insights into ecosystem functioning and resilience [7, 16]. Additionally, understanding insect behaviour and interactions during decomposition has potential applications in medical research, such as wound healing and forensic pathology [92, 93], as well as understanding how different species can coexist on limited and patchy ephemeral resources in nature [94, 95].

Although there was no clear trend observed in the year of publication for entomology and decomposition, there has been an increasing prevalence of research in this field over the last decade. This trend indicates the ongoing interest and importance of studying insect behaviour and their role in decomposition processes.

### Insects (pre-appearance interval (PAI) and oviposition)

Understanding the duration and factors influencing the PAI is crucial in forensic investigations as it helps estimate the minimum post-mortem interval (PMI_min_), which can be useful when determining the time of death [51]. Despite the significance of PAI and colonisation behaviour as key variables in the decomposition timeline, they have received limited attention in the literature [96, 97]. Investigating the impact of PAI and oviposition on decomposition rates, microbial succession, and VOC production can reveal their significance as drivers of variation in decay. Olfaction plays a significant role in the attraction of necrophagous insects and their subsequent colonisation of remains. VOCs, which partly come from the corpse or from endogenous and exogenous bacteria surrounding it or from the carcass itself, are released as remains decay and are what give them their distinctive smell [98]. An investigation by [99] discovered that cadavers concealed within tents significantly impacted colonisation time of flies; this ultimately led to a prolonged PAI and could lead to an under-estimation of the PMI_min_. This also resulted in an overall retarded decomposition rate [99]. It is also important to note that toxicants may also directly influence the PAI of insects. For example, an earlier research study conducted by the author [50] determined that the addition of hydrated lime and bleach to remains post-mortem resulted in a significantly longer PAI (> 12 h) and delayed oviposition. By incorporating these factors into decomposition studies, researchers can gain a more comprehensive understanding of the complex ecological processes occurring during decomposition.

Although most publications in our review focused on forensic science, our review identified two articles that encompassed a combined discipline of forensic and ecology. This suggests a recognition of the ecological aspects associated with PAI and insect colonisation/oviposition. Ecology plays a vital role in understanding the interactions between insects and their environment, including the factors that influence their colonisation patterns and oviposition behaviour [100]. Considering the ecological aspects of insect activity during decomposition can provide insights into the broader ecological processes and ecosystem dynamics. The number of articles focused on PAI and oviposition in relation to organic decomposition has been slowly increasing in recent years. This indicates a growing recognition of the importance of studying the timing and patterns of insect colonisation on remains.

### Volatile Organic compounds (VOCs)

Further investigation is warranted to explore the relationship between VOCs and other factors that contribute to variations in the decomposition process [101–104]. . The absence of research specifically exploring the impact of drugs and toxins on VOC production during decomposition is a notable gap, as none of our reviewed literature within this topic gave any consideration to the effect of these chemicals on VOCs. The production of VOCs is closely tied to microbial activity, as these compounds are generated as by-products of microbial processes [105]. Additionally, the presence of drugs or toxins can have a significant impact on the microbial profile, potentially altering the composition and activity of microorganisms involved in decomposition processes and subsequent VOC production [106]. Therefore, it is a notable gap that our review did not evaluate any articles on this topic. Studying the interactions between drugs and toxins, microbial activity, insect behaviour, and VOC profiles can provide valuable insights into the forensic implications and ecological consequences of drug presence in decomposition scenarios [19]. For example, a study by [107] demonstrated that the VOCs released by decaying remains are influenced by microbial activity and contribute to attracting insects to the decaying matter. These VOCs serve as a signal to insects, indicating the presence of a transient and valuable resource [108].

The majority of publications on VOCs in decomposition science were within a forensic science discipline, emphasising their significance in forensic investigations for estimating the PMI. However, the recognition of ecological implications in some publications and the presence of articles solely within the ecology discipline demonstrate the potential for interdisciplinary research in understanding the broader ecological context of VOC emissions during decomposition [109–112]. Integrating ecological perspectives into VOC research can enhance our understanding of the ecological implications of decomposition and contribute to a more comprehensive understanding of ecosystem dynamics.

Publications on VOCs related to decomposition started in 2005, peaked in 2015 with 10 articles, and have shown a gradual increase in recent years. This indicates a sustained interest in studying the chemical cues emitted by decomposing remains and their role in attracting insects. VOCs play a crucial role in the detection and attraction of insects to decaying matter, highlighting the importance of understanding the chemical ecology of decomposition.

### Drugs and toxins

Drugs and toxins can have a significant effect on the decomposition process and the interpretation of forensic evidence [50]. The presence of drugs (prescribed or illicit) or other toxins in a deceased individual’s system can influence the rate and pattern of decomposition, yet these effects are largely unknown, leading to unquantifiable errors in PMI estimations [113]. The use of human cadavers in forensic research has steadily increased over the years through the advent of outdoor human taphonomy facilities [114]. However, there is limited research which investigates or considers the effect of peri-mortem treatments on cadaver decomposition, despite the availabilities of such facilities, and evidence which suggests these intrinsic factors introduce variability in decay rates [18, 60, 115]. Certain chemicals, such as antibiotics or preservatives, is intended to inhibit microbial activity in living individuals but may have subsequent impacts on the bouquet of VOCs released from a body after death, although there is no current literature to support this idea yet [116]. On the other hand, there are substances such as illicit and prescription drugs, which can accelerate the decomposition process through various mechanisms [117]. Understanding the effects of drugs and other toxins on decomposition can help forensic scientists accurately estimate the time since death and interpret decomposition patterns in medicolegal investigations.

The distribution of scientific disciplines in drug and other toxin related articles pertaining to decomposition science offers valuable insights into the research focus and practical applications within this field. Our review identified that most of the reviewed articles (12) were within the forensic science discipline. Forensic science plays a crucial role in determining the cause and circumstances of death, estimating the PMI, and providing evidence for legal proceedings [50, 93, 118, 119]. The inclusion of drug and toxin research in forensic contexts reflects the importance of understanding the effects of these chemicals on decomposition processes [2, 37, 50]. By examining drugs and other toxins in decomposition, forensic scientists can enhance their ability to accurately interpret decomposed remains and provide more precise forensic analyses based on decomposition rates and toxin half-lives [49, 51, 58, 59].

Additionally, a few articles were categorised under other combination fields of forensic/veterinary (1) and forensic/medicine (3). These interdisciplinary approaches demonstrate the recognition of the broader applications of drugs and toxins research in decomposition science. Veterinary forensic science investigates animal deaths and crimes [93, 120], while forensic medicine focuses on the application of medical knowledge to legal issues. The inclusion of drugs and other toxins aspects in these fields suggests the importance of understanding the role of these chemicals in the decomposition of animal remains and in cases involving medical contexts, such as drug-related deaths or medical malpractice [121]. The limited representation of drug and toxin studies in ecological or medical contexts suggests potential areas for future research. Exploring the ecological impacts of pharmaceutical residues or other high profile chemical contaminants (e.g., PFAS) during decomposition or investigating the effects of drugs on other DoV such as the microbiome, insect behaviour and VOCs, could provide valuable insights into broader ecological and medical implications.

There does not appear to be a clear trend in the publication of articles related to drugs and other toxins in decomposition science. The number of publications in this area has been relatively stable, with no significant increase or decrease over the years. While not exhibiting a notable trend, the consistent publication of articles indicates the ongoing interest in understanding the interaction between the effect of drugs and toxins and decomposition.

### Multidisciplinary approach to decomposition research

Collaboration among researchers specialising in various aspects of decomposition science is essential for bridging the gap between scientific disciplines and achieving a more holistic understanding of the field. By fostering interdisciplinary collaboration between experts in microbial ecology, entomology, chemistry, pharmacology, and forensic science, we can explore the synergistic effects, feedback loops, and complex interactions between the drivers of variation in decay. Forensic science is crucial to the administration of justice, with a call to aid being issued by many researchers, for the larger scientific community to advocate for more collaborative research, which is systematic, reliable, and affordable [119]. This has also been encouraged to extend beyond a forensic application, and to also be applied to other areas of decomposition research, such as ecology [122]. Our review highlights missed opportunities in a multidisciplinary approach to decomposition science due to a clear gap in the literature where a variety of DoV are studied. The authors have in a previous article highlighted how to bridge this gap in one-dimension, however, the evidence shows that there is more to be done in a multidisciplinary space considering a multitude of DoV [5]. Multidisciplinary studies will assist in providing a comprehensive understanding of decomposition science, which will not only enhance forensic investigations but also contribute to ecological research and conservation efforts by uncovering the ecological processes underlying nutrient recycling and ecosystem functioning.

## Implications and conclusions

Our findings underscore the need to improve understanding of the interconnectedness of different drivers of variation in decay and their collective impact on decomposition processes. Although our study is not an exhaustive review of all literature surrounding decomposition science, it provides a foundation for future research and supports the need for decomposition science to incorporate greater sophistication into their design, such as building on the necrobiome concept or other multidisciplinary models. Such research is needed to investigate the relationships between microbes, insects, PAI and oviposition, VOCs, and drugs/toxins, with an emphasis on collaborative efforts within the decomposition science community. By collaborating across multiple disciplines, we can advance our understanding of decomposition science and contribute to a more comprehensive and holistic perspective of this crucial ecological process.
